# Schwerpunkt künstliche Intelligenz in der Medizin – rechtliche Aspekte bei der Nutzung großer Sprachmodelle im klinischen Alltag

**DOI:** 10.1007/s00108-025-01861-0

**Published:** 2025-03-14

**Authors:** Eva Weicken, Mirja Mittermaier, Thomas Hoeren, Juliana Kliesch, Thomas Wiegand, Martin Witzenrath, Miriam Ballhausen, Christian Karagiannidis, Leif Erik Sander, Matthias I. Gröschel

**Affiliations:** 1 Fraunhofer-Institut für Nachrichtentechnik, Heinrich-Hertz-Institut, Einsteinufer 37, 10587 Berlin, Deutschland; 2 Fächerverbund für Infektiologie, Pneumologie, und Intensivmedizin, Charité – Universitätsmedizin Berlin, Südring 9, 13353 Berlin, Deutschland; 3 Berlin Institute of Health at Charité, Anna-Louisa-Karsch-Str. 2, 10178 Berlin, Deutschland; 4 Institut für Informations‑, Telekommunikations- und Medienrecht (ITM), Universität Münster, Leonardo-Campus 9, 48149 Münster, Deutschland; 5 Bird & Bird LLP, Am Sandtorkai 50, 20457 Hamburg, Deutschland; 6 Technische Universität Berlin, Straße des 17. Juni 135, 10623 Berlin, Deutschland; 7 Lungenklinik Köln-Merheim, Abteilung für Pneumologie und Intensivmedizin, ARDS and ECMO Zentrum, Ostmerheimer Str. 200, 51109 Köln, Deutschland; 8 Universitätsklinikum Witten/Herdecke, Alfred-Herrhausen-Straße 50, 58455 Witten, Deutschland

**Keywords:** Künstliche Intelligenz/Rechtsgutachten, Natürliche Sprachverarbeitung, Große Sprachmodelle/klinische Implementierung, Cloud Computing, Datenschutz, Artificial intelligence/legal opinion, Natural language processing, Large language models/clinical implementation, Cloud computing, Data protection

## Abstract

**Hintergrund:**

Die Nutzung von künstlicher Intelligenz (KI) und Methoden der natürlichen Sprachverarbeitung (NLP) in der Medizin, insbesondere von großen Sprachmodellen (LLM), bietet Möglichkeiten, das Gesundheitssystem und die Patientenversorgung in Deutschland voranzubringen. LLM haben zuletzt an Bedeutung gewonnen, jedoch ist ihre praktische Anwendung in Kliniken und Praxen bisher begrenzt. Erforschung und Implementierung werden durch eine komplexe Rechtslage gehemmt. Es ist essenziell, LLM in klinischen Studien in Deutschland zu erforschen und an den gesetzlichen Rahmen angepasste Anwenderleitlinien zu entwickeln.

**Ziel der Arbeit:**

Wie können wir Grundlagen für die datenschutzkonforme Nutzung von LLM, insbesondere von Cloud-basierten LLM, im deutschen Gesundheitssystem schaffen? In der vorliegenden Arbeit sollen die datenschutzrechtlichen Aspekte der Nutzung Cloud-basierter LLM in der klinischen Forschung und Patientenversorgung in Deutschland und der Europäischen Union (EU) dargestellt werden; Kernaussagen eines Rechtsgutachtens hierzu werden betrachtet. Soweit die Nutzungsanforderungen in Landesgesetzen geregelt sind, wird auf die Rechtslage in Berlin abgestellt.

**Material und Methoden:**

Im Rahmen eines Forschungsprojekts wurde ein Rechtsgutachten in Auftrag gegeben, um die datenschutzrechtlichen Aspekte der Verwendung von LLM mit Cloud-basierten Lösungen an der Charité – Universitätsmedizin Berlin zu klären.

**Ergebnisse:**

Die rechtlichen Rahmenbedingungen variieren je nach Art der Datenverarbeitung und teilweise je Bundesland. Bei anonymen Daten sind datenschutzrechtliche Anforderungen generell nicht einschlägig. Soweit personenbezogene Daten verarbeitet werden, sollten diese nach Möglichkeit pseudonymisiert werden. Im Forschungskontext ist im Regelfall eine Einwilligung der PatientInnen notwendig, um deren personenbezogene Daten zu verarbeiten. Es müssen Auftragsverarbeitungsvereinbarungen mit den Anbietern geschlossen werden. Die von LLM stammenden Empfehlungen müssen stets ärztlich überprüft werden.

**Schlussfolgerung:**

Die Nutzung Cloud-basierter LLM ist möglich, solange Datenschutzanforderungen beachtet werden. Die rechtlichen Rahmenbedingungen sind komplex und erfordern von Anbietern Transparenz. Zukünftige Entwicklungen könnten das Potenzial von KI und LLM im Speziellen im Klinikalltag erhöhen, jedoch sind klare rechtliche und ethische Vorgaben notwendig.

## Abstract (en)

**Background:**

The use of artificial intelligence (AI) and natural language processing (NLP) methods in medicine, particularly large language models (LLMs), offers opportunities to advance the healthcare system and patient care in Germany. LLMs have recently gained importance, but their practical application in hospitals and practices has so far been limited. Research and implementation are hampered by a complex legal situation. It is essential to research LLMs in clinical studies in Germany and to develop guidelines for users.

**Objective:**

How can foundations for the data protection-compliant use of LLMs, particularly cloud-based LLMs, be established in the German healthcare system? The aim of this work is to present the data protection aspects of using cloud-based LLMs in clinical research and patient care in Germany and the European Union (EU); to this end, key statements of a legal opinion on this matter are considered. Insofar as the requirements for use are regulated by state laws (vs. federal laws), the legal situation in Berlin is used as a basis.

**Materials and methods:**

As part of a research project, a legal opinion was commissioned to clarify the data protection aspects of the use of LLMs with cloud-based solutions at the Charité – University Hospital Berlin, Germany. Specific questions regarding the processing of personal data were examined.

**Results:**

The legal framework varies depending on the type of data processing and the relevant federal state (*Bundesland*). For anonymous data, data protection requirements need not apply. Where personal data is processed, it should be pseudonymized if possible. In the research context, patient consent is usually required to process their personal data, and data processing agreements must be concluded with the providers. Recommendations originating from LLMs must always be reviewed by medical doctors.

**Conclusions:**

The use of cloud-based LLMs is possible as long as data protection requirements are observed. The legal framework is complex and requires transparency from providers. Future developments could increase the potential of AI and particularly LLMs in everyday clinical practice; however, clear legal and ethical guidelines are necessary.

Künstliche Intelligenz (KI) und natürliche Sprachverarbeitung („natural language processing“ [NLP]), beispielsweise die großen Sprachmodelle („large language models“ [LLM]) GPT‑4 von OpenAI [1] und Bard [2] oder PaLM 2 [3] von Google sowie die großen multimodalen Modelle („large multimodal models“ [LMM]), wie Gemini von Google [4], bieten viele innovative Einsatzmöglichkeiten in der Medizin. Diese erstrecken sich neben Alltagsanwendungen vor allem auf das Gesundheitswesen [5], etwa die Verarbeitung und das Verständnis klinischer Dokumentation [6] oder die Formulierung patientenspezifischer Antworten auf Anfragen [7] bis hin zu Avataren, die bereits heute in Ländern wie Israel zur Ersteinschätzung im Notfall eingesetzt werden.

Es ist anzunehmen, dass LLM bei der Digitalisierung des Gesundheitswesens in den kommenden Jahren eine relevante Rolle spielen werden [8]. Diese Annahme spiegelt sich unter anderem in der Digitalstrategie der Bundesregierung wider, welche die Entwicklung und Anwendung digitaler Technologien im Gesundheitssektor ressortübergreifend vorantreibt [9]. Das Bundesministerium für Gesundheit (BMG) erarbeitet für die Umsetzung der Digitalstrategie derzeit verschiedene Gesetze [10]. Akzeptanz und Wahrnehmung des Einsatzes von KI-Modellen in der Medizin sowohl durch PatientInnen als auch durch Gesundheitsdienstleister werden als zentrale Faktoren für die Wirksamkeit und Einsatzfähigkeit digitaler Lösungen in Gesundheitseinrichtungen gesehen [11]. Das wirtschaftliche Potenzial dieser digitalen Lösungen ist gerade in Zeiten zunehmender Deindustrialisierung enorm.

Klinische Forschungsvorhaben mit großen Sprachmodellen wurden in Deutschland bisher kaum umgesetzt

Was aber kommt von diesem Potenzial digitaler Lösungen im Klinikalltag an? Die konkrete Umsetzung und praktische Anwendung dieser Technologien – einschließlich LLM in der klinischen Versorgung – ist bislang ernüchternd [12]. Klinische Forschungsvorhaben mit LLM sind bisher in Deutschland kaum umgesetzt worden [13]. Um den Wert von LLM für den deutschen Kontext auch unter europäischen Datenschutzaspekten zu evaluieren, ist es wichtig, die Evaluation auch lokal durchzuführen. Dem steht als Barriere die komplexe und zwischen den Bundesländern leicht abweichende rechtliche Lage hinsichtlich der Nutzung LLM- und Cloud-basierter Anwendungen im Gesundheitssektor entgegen. Der Vorteil LLM- und Cloud-basierter Anwendungen besteht darin, dass ein jederzeit einfach über das Internet zugänglicher und gemeinsam nutzbarer Pool von Rechenressourcen, unter anderem mit Servern, Netzwerken, Speichern und Anwendungen, zur Verfügung steht, ohne dass vorab Investitionen in die lokale Infrastruktur notwendig sind [14]. Ein vom BMG gefördertes Studienvorhaben mit GPT‑4 von OpenAI an der Charité – Universitätsmedizin Berlin veranlasste die AutorInnen, ein Rechtsgutachten diesbezüglich einzuholen. Ziel des vorliegenden Beitrags ist es, die Kernaussagen dieses Gutachtens zur Nutzung Cloud-basierter LLM darzustellen.

## Was sind LLM und Cloud-basierte Lösungen?

LLM werden innerhalb des breiten Felds der KI der Kategorie des NLP zugeordnet [15]. Bisherige KI-Modelle wurden für eine einzelne und spezifische Aufgabe, meist auf einem annotierten und aufwendig händisch klassifizierten Datensatz, entwickelt und trainiert, beispielsweise zur Pneumoniedetektion auf Röntgenbildern [16] oder zur Textverarbeitung. Diese Methode erfordert zunächst das Trainieren des Modells auf großen Testdatensätzen anhand von Zehntausenden von Beispielen für eine spezifische Aufgabe, wodurch die Leistung des Modells stetig verbessert wird [17]. Die Weiterentwicklung dieser NLP-Methoden in den letzten Jahren mithilfe immer größerer Datenmengen aus dem Internet macht es nun möglich, komplexe Inhalte zu generieren (man spricht auch von generativer KI), Aufgaben zu lösen und mit NutzerInnen zu interagieren. Googles medizinspezifisches LLM Med-PaLM 2 beantwortete beispielsweise Multiple-Choice-Fragen im amerikanischen Medizinexamen „United States Medical Licensing Examination“ (USMLE) zu 85 % korrekt, vergleichbar mit der Leistung von ÄrztInnen [18].

Im Gesundheitssektor haben LLM das Potenzial, durch ihre vielfältigen Einsatzmöglichkeiten und Fähigkeiten den Klinikalltag nachhaltig zu erleichtern. Beispiele hierfür sind die Unterstützung beim Verfassen medizinischer und pflegerischer Dokumentation, die Vereinfachung und Effizienzsteigerung von Abrechnungsprozessen und administrativen Aufgaben sowie die effizientere Nutzung von Leitlinien durch die Fähigkeit der LLM, große Mengen an unstrukturiertem Text zu strukturieren [7, 19]. Die automatisierte Brieferstellung mithilfe von KI wird bereits jetzt in der Praxis durchgeführt [20].

Vor dem Hintergrund dieser rasanten Weiterentwicklung von KI und LLM mit immer weitreichenderen Funktionen, wie der Unterstützung klinischer Entscheidungsprozesse („clinical agent“; [21]), ist es entscheidend, über gesetzliche, regulatorische, aber auch praktische Maßnahmen zu informieren, um Aufklärung und Akzeptanz bei den Nutzenden zu steigern. Zukünftige Trends großer Softwareunternehmen gehen in Richtung einer Weiterentwicklung multimodaler LMM (diese verarbeiten nicht nur Text, sondern auch Bilder sowie Audio- und Videodaten, um Informationen [noch] effizienter zu finden, kreative Aufgaben zu bewältigen und natürliche Stimmen zu erzeugen) oder einer Weiterentwicklung von Open-Source-Modellen (Google [4], Mistral AI [22]).

**Abb. 1:**
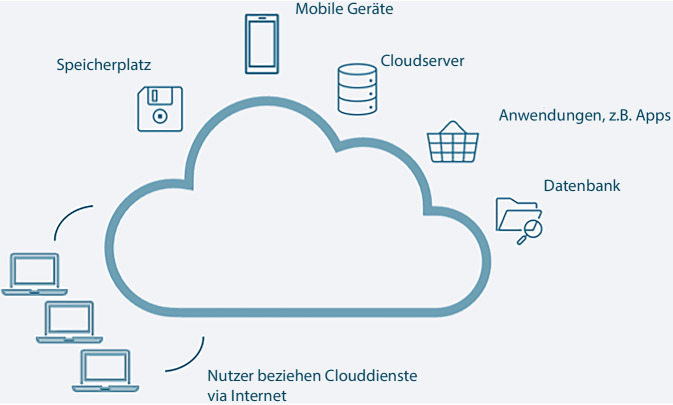
Schematische Darstellung des Cloud Computing. „Cloud“ (englisch für Wolke) bezeichnet eine informationstechnische (IT) Infrastruktur, die sich auf mehrere Rechner verteilt und deren physische Hardware für den Nutzer unsichtbar bleibt. Sowohl Unternehmen als auch Privatpersonen können Cloud-Dienste als Speicher oder zur Bereitstellung komplexer IT-Services nutzen, meist über das Internet

Die Fähigkeiten, neue Inhalte ohne aufwendiges Training zu erzeugen, unterscheiden LLM von bisherigen KI-Modellen. Dies erfordert große Serverkapazitäten, wie sie meist von kommerziellen Anbietern wie Microsoft Azure oder Google angeboten werden. Diese Bereitstellung von Speicherplatz und diversen informationstechnischen (IT) Diensten auf einem großen Server mit einfachem Zugang und Nutzung über das Internet wird den sogenannten Cloud-Computing-basierten Lösungen zugeordnet (Infobox 1 und schematische Darstellung in Abb. 1 zu Cloud Computing).

### Infobox 1 Cloud Computing: Beschreibung der Bedeutung [23] und Funktion von Cloud Computing

Beschreibung:

- Der Begriff „Cloud Computing“ ist bisher nicht allgemeingültig definiert.
- Die Definition der International Organisation of Standardisation (ISO) hat Cloud Computing in einer Norm definiert: „Paradigma, einen netzwerkbasierten Zugang auf ein skalierbares und elastisches Reservoir gemeinsam nutzbarer physischer oder virtueller Ressourcen nach dem Selbstbedienungsprinzip und bedarfsgerechter Administration zu ermöglichen.“[24]

Vorteile:

- Die Vorteile eines Cloud-Computing-basierten Gesundheitssystems bestehen beispielsweise in der Förderung der Interoperabilität, einer flexiblen Nutzung und Skalierbarkeit.

Herausforderungen und Risiken:

- Diese betreffen beispielsweise die Einhaltung der Privatsphäre und Datensicherheit in der Cloud oder Schwierigkeiten der Integration in bestehende informationstechnische (IT) Systeme.

## Anwendungen großer Sprachmodelle in der Medizin

Die Anwendbarkeit von KI, einschließlich LLM, im Gesundheitswesen hängt in großem Maße vom Grad der Digitalisierung des Gesundheitssystems, der vorhandenen IT-Infrastruktur, qualifizierten Fachkräften, der Organisation und vor allem dem Zugriff auf hochaufgelöste Daten ab. Dies ist in der Realität herausfordernd, unter anderem aufgrund

- der sektoral gegliederten Krankenhausstruktur,
- veralteter Krankenhausinformationssysteme (KIS),
- einer fehlenden einheitlichen Governance-Struktur in Krankhäusern,
- einer teils schlechten technischen Ausstattung [25] und vor allem
- einer unterschiedlichen Semantik.

Diese Faktoren erschweren eine effektive digitale Anschlussfähigkeit und Interoperabilität stationärer und ambulanter Gesundheitseinrichtungen und führen somit zu einem langsamen Fortschreiten der Digitalisierung in Deutschland. Laut dem Electronic-Medical-Records-Adoption-Model-Score [26], der den Grad der Digitalisierung in Krankenhäusern auf sieben Ebenen international vergleicht, besteht in Deutschland Aufholbedarf [14]. Die Vorteile digitaler Medizin und digitaler Ökosysteme liegen nicht nur in der Effizienzsteigerung durch Prozessvereinfachung – beispielsweise durch Diagnosis-related-group(DRG)-Manager und papierlose Dokumentation, wobei LLM durch ihre Fähigkeit, unstrukturierten in strukturierten Text umzuwandeln, eine wichtige Rolle spielen –, sondern auch in der Erweiterung der Therapieoptionen und des Patientenmanagements durch personalisierte Medizin. Digitale Ökosysteme nutzen üblicherweise Cloud-basierte Lösungen und werden in der Medizin bereits in einigen Bereichen erfolgreich verwendet [14]: in kardiologischen Telemedizinservices für die Analyse von Elektrokardiogrammen [27], zur digitalisierten Bildanalyse in der Onkologie [28] sowie zur Entscheidungsunterstützung für geeignete Therapien [29] und bei der Dosiskalkulation für komplizierte therapeutische Verfahren in der Radiotherapie [30].

Die Implementierung und Nutzung Cloud-basierter Lösungen in der Medizin ermöglicht den Aufbau eines dezentralen, interoperablen Ökosystems [31]. Die flächendeckende Implementierung und Nutzung Cloud-basierter Lösungen im Einklang mit datenschutzrechtlichen und rechtlichen Bestimmungen bleibt jedoch herausfordernd. Putzier et al. [14] stellen anhand des konkreten Beispiels eines großen deutschen Universitätsklinikums Strategien zur Einführung Cloud-basierter Lösungen vor, die den Anforderungen deutscher, europäischer und internationaler Richtlinien gerecht werden (auf europäischer Ebene ist hier insbesondere die Datenschutz-Grundverordnung [DSGVO] zu nennen, eine Verordnung der Europäischen Union [EU] zur Stärkung und Vereinheitlichung des Datenschutzes für Individuen in der EU und im Europäischen Wirtschaftsraum). Der Nutzen Cloud-basierter LLM, wie ChatGPT, liegt beispielsweise in der einfachen Implementierung und Handhabung mit Zugriff über das Internet. Die hohe Rechenleistung, wie sie bei großen Cloud-Anbietern gegeben ist, ermöglicht die Verarbeitung großer Datenmengen und Berechnung komplexer Aufgaben in kurzer Zeit.

## Komplexe rechtliche Situation hinsichtlich der Nutzung großer Sprachmodelle im Gesundheitswesen – ein Lösungsansatz

Klinische Forschungsvorhaben mit LLM beinhalten eine umfangreiche Klärung des ethischen und rechtlichen, insbesondere datenschutzrechtlichen Rahmens der Nutzung von LLM im Forschungskontext und in der Patientenversorgung. Ein konkretes Beispiel stellt ein Forschungsvorhaben der Charité – Universitätsmedizin Berlin dar, das den Einsatz von LLM mit einer Cloud-basierten Lösung auf einer internistischen Station beinhaltet. Die Forschungsgruppe sah sich mit einer unklaren rechtlichen Situation konfrontiert. Daher sollte in einem Pilotprojekt eine Grundlage für Cloud-basierte LLM-Forschungsprojekte geschaffen werden, in Form eines Ethikvotums und eines Rechtsgutachtens anhand eines konkreten Beispiels und klinischen Studienvorhabens.

Im Rahmen des genannten Pilotprojekts erstellte eine Kanzlei ein Rechtsgutachten, um die Fragen zur Nutzung von LLM mit einer Cloud-basierten Lösung für dieses spezifische Pilotprojekt zu untersuchen. Hierbei wurden unter anderem folgende Fragen adressiert:

- Welche datenschutzrechtlichen Aspekte sind von der Charité in Bezug auf die Nutzung Cloud-basierter Lösungen im klinischen Forschungskontext zu beachten?
- Welche Daten dürfen von den Anbietern solcher Cloud-basierter Lösungen im Forschungskontext verarbeitet werden?

Die Erkenntnisse, die die AutorInnen des vorliegenden Beitrags aus dem Rechtsgutachten gezogen haben, werden im Folgenden beschrieben. Dabei übernehmen die AutorInnen dieses Beitrags keine Garantie, dass die beschriebenen Erkenntnisse in jedem Fall korrekt dem Gutachten entnommen wurden.

Zusammenfassend lässt sich sagen, dass sich die rechtlichen Bedingungen unterscheiden, je nachdem ob es sich um die Nutzung anonymer (nichtpersonenbezogener) Daten oder die Verarbeitung personenbezogener Daten handelt. Wenn mit der Cloud-basierten Lösung lediglich anonyme Daten verarbeitet werden, sind bei dieser Datenverarbeitung keine datenschutzrechtlichen Anforderungen zu erfüllen. Wenn mit der Cloud-basierten Lösung personenbezogene Daten verarbeitet werden, gilt – in Berlin – insbesondere Folgendes (Zusammenfassung siehe Infobox 2):

- Falls keine Anonymisierung der personenbezogenen Daten möglich ist, sollten sie – soweit machbar – derart pseudonymisiert werden, dass der Anbieter keinen Bezug zu den einzelnen Personen herstellen kann. Zudem dürfen nur solche Daten verarbeitet werden, die zweckgebunden relevant und erforderlich sind.
- Im Forschungskontext ist bei der Verarbeitung personenbezogener Daten durch die Cloud-basierte Lösung regelmäßig die Einwilligung der PatientInnen notwendig. Bei der Verarbeitung klinischer Routinedaten kann man häufig argumentieren, dass auf eine Einwilligung verzichtet werden kann.
- Mit dem LLM- bzw. Cloud-Anbieter muss eine Auftragsverarbeitungsvereinbarung geschlossen werden. Wenn der Anbieter die personenbezogenen Daten, die er im Auftrag des Auftraggebers, beispielsweise eines Krankenhauses, verarbeitet, dazu verwendet, seine Cloud-basierte Lösung zu trainieren, muss hierfür (zusätzlich) die Einwilligung der PatientInnen eingeholt werden. Zudem bestehen in diesem Fall unter anderem erhöhte Transparenzpflichten gegenüber den PatientInnen, und es besteht ein erhöhtes Haftungsrisiko des Auftraggebers.
- Falls dem LLM- bzw. Cloud-Anbieter ein Rückschluss auf die Identität einzelner Personen möglich ist, muss die Datenverarbeitung (1) in einem Land des Europäischen Wirtschaftsraums oder (2) in einem Land, für das ein Angemessenheitsbeschluss der EU-Kommission besteht, erfolgen oder (3) durch angemessene Garantien flankiert werden. Zu Letzteren zählt beispielsweise der Abschluss sogenannter Standardvertragsklauseln mit dem Anbieter. Je nach Bundesland, in dem etwa das betreffende Krankenhaus sitzt, können andere Anforderungen betreffend den Verarbeitungsstandort bestehen.
- Die abschließende Entscheidung über die Behandlungsansätze muss von ärztlicher Seite erfolgen. Die Empfehlungen der Cloud-basierten Lösung müssen also stets einer menschlichen Überprüfung unterzogen werden.
- Die Datenschutzerklärung des Auftraggebers muss die Beteiligung des LLM- bzw. Cloud-Anbieters an der Datenverarbeitung widerspiegeln. Zudem sollte der Auftraggeber möglichst eine Datenschutzfolgeabschätzung für den Einsatz der Cloud-basierten Lösung durchführen.
- Es fällt auf, dass bei einigen großen Cloud-Anbietern komplexe Produktinformationen und Vertragsbedingungen eine abschließende rechtliche Bewertung erschweren.

Auf Basis des Rechtsgutachtens scheint die Nutzung Cloud-basierter LLM daher möglich, solange die grundlegenden Datenschutzanforderungen beachtet werden. Hierzu sind häufig auch verbindliche Zusagen der LLM- bzw. Cloud-Anbieter zum Verarbeitungs- und Serverstandort hilfreich bzw. erforderlich, außerdem die Zusage, dass Daten nicht gespeichert oder für das Trainieren des LLM genutzt werden. Dies sollte sich aus den Vertragsunterlagen bzw. Produktbeschreibungen des Cloud-Anbieters ergeben oder anderweitig dokumentiert zugesichert werden. Falls vonseiten des Anbieters keine Zusage zum Verarbeitungs- und Serverstandort gegeben wird, empfehlen wir, auf andere Cloud- und LLM-Anbieter auszuweichen.

Die rechtlichen Bedingungen hängen davon ab, ob die verarbeiteten Daten personenbezogen sind

Insgesamt besteht die rechtliche Einschätzung, dass sich LLM hinsichtlich ihrer Nutzung im Forschungskontext nicht von anderen Tools zur Datenverarbeitung unterscheiden.

### Infobox 2 Kernaussagen zu rechtlichen Grundlagen – Zusammenfassung der wichtigsten rechtlichen Rahmenbedingungen in Berlin für die Nutzung von KI, inklusive LLM, sowie generell für die Verarbeitung auch patientenbezogener Daten im klinischen Kontext

Bei der Nutzung anonymer Daten sind keine datenschutzrechtlichen Anforderungen zu erfüllen.

Bei der Verarbeitung personenbezogener Daten gilt:

- Falls keine Anonymisierung möglich ist, sollten Daten soweit machbar pseudonymisiert und nur zweckgebunden in der Cloud verarbeitet werden.
- Im Forschungskontext ist eine Patienteneinwilligung notwendig.
- Mit dem Anbieter muss eine Auftragsverarbeitungsvereinbarung geschlossen werden.
- Falls ein Rückschluss auf die Identität einzelner Personen durch den LLM- bzw. Cloud-Anbieter möglich ist, muss die Datenverarbeitung
  - in einem Land des Europäischen Wirtschaftsraums oder
  - in einem Land, für das ein Angemessenheitsbeschluss der EU-Kommission besteht, erfolgen oder
  - durch angemessene Garantien flankiert werden (beispielsweise Abschluss sogenannter Standardvertragsklauseln mit dem Anbieter und je nach Bundesland gegebenenfalls weitere Anforderungen den Verarbeitungsstandort betreffend).
- Die abschließende Entscheidung über die Behandlungsansätze muss von ärztlicher Seite erfolgen (menschliche Überprüfung).
- Die Datenschutzerklärung des Auftraggebers muss die Beteiligung des LLM- bzw. Cloud-Anbieters an der Datenverarbeitung widerspiegeln. Zudem sollte möglichst eine Datenschutzfolgeabschätzung für den Einsatz der Cloud-basierten Lösung durchgeführt werden.
- Zu beachten: Komplexe Produktinformationen und Vertragsbedingungen einiger großer Cloud-Anbieter erschweren eine abschließende rechtliche Bewertung.

*EU* Europäische Union, *KI* künstliche Intelligenz, *LLM* „large language model“ (großes Sprachmodell)

## Fazit für die Praxis

- Vor uns liegt eine spannende Zukunft mit dem Durchbruch diverser Anwendungen künstlicher Intelligenz (KI) im Klinikalltag. Erst wenn dies an der PatientIn erlebbar wird, lässt sich der Mehrwert von KI und großen Sprachmodellen (LLM) realisieren.
- Im Idealfall sprechen PatientInnen zur Ersteinschätzung vor dem Arztgespräch in Zukunft zunächst mit einem Avatar, und nichtinvasive Sensoren geben binnen Sekunden erste klinische Anhaltspunkte über PatientInnen.
- Die Erforschung von LLM im klinischen Umfeld ist herausfordernd, aber prinzipiell möglich. Das Potenzial von LLM sollte auch in Deutschland erforscht werden.
- Die vertrauensvolle Anwendung erfordert neben der Einbindung multidisziplinärer Stakeholder klare rechtliche und ethische Rahmenbedingungen und klinische Evidenz sowie eine rigorose Evaluation durch klinische Studien.
- Eine Nutzung Cloud-basierter LLM ist bei Verwendung anonymisierter Daten ohne datenschutzrechtliche Beschränkungen möglich. Auch bei Eingabe personenbezogener Daten kann sie zulässig sein. Jedoch sollte jedes Vorhaben individuell geprüft und abgewogen werden.
- Es sollte darauf hingearbeitet werden, dass die notwendigen Schritte hinsichtlich einer Transparenzsteigerung unternommen werden, um die Potenziale Cloud-basierter LLM-Lösungen voll ausschöpfen zu können.
